# Human leptospirosis in Tanzania: sequencing and phylogenetic analysis confirm that pathogenic *Leptospira* species circulate among agro-pastoralists living in Katavi-Rukwa ecosystem

**DOI:** 10.1186/s12879-016-1588-x

**Published:** 2016-06-10

**Authors:** Shabani K. Muller, Justine A. Assenga, Lucas E. Matemba, Gerald Misinzo, Rudovick R. Kazwala

**Affiliations:** Sokoine University of Agriculture, Morogoro, Tanzania; National Institute for Medical Research (NIMR), MOMS clinical Trial, Morogoro, Tanzania

**Keywords:** Leptospirosis, Sequencing, Phylogenetic Analysis, Katavi-Rukwa Ecosystem, Tanzania

## Abstract

**Background:**

Leptospirosis is a neglected zoonotic disease of worldwide public health importance. The disease affects humans, domestic animals and wildlife. However, leptospirosis is challenging in its diagnosis in humans. Culture technique, which is time consuming, is not recommended for clinical diagnosis. For these reasons, serological and molecular techniques remain the test of choice. The major objective of this study was to explore the genetic characteristic of *Leptospira* species which are prevalent among agro-pastoralists living in Katavi–Rukwa Ecosystem, Tanzania.

**Methods:**

A cross-sectional epidemiological study was carried out in the Katavi-Region South-west, Tanzania between August, 2013 and November, 2014. A total of 267 participants were randomly recruited for the study. Microscopic agglutination test (MAT) was used to detect antibody against six *Leptospira* antigens including local serogroups Icterohaemorrhagiae, Ballum, Grippotyphosa, Sejroe and reference serogroups Hebdomadis, and Australis. Samples with MAT titers ≥ 1:160 were scored as positive, samples with MAT titers ranging from 1:20 to 1:80 were scored as exposed to *Leptospira,* and absence of agglutination titers was scored as negative. All MAT positive samples, including the low titre samples were subjected to PCR using the respective 16S rRNA primers for the pathogenic and non-pathogenic species.

**Results:**

Out of 267 samples tested, 80 (29.9 %) were positive with MAT. The major circulating leptospiral serogroups were Sejroe (15.7 %,), Icterohaemorrhagiae (8.9 %), Grippotyphosa (4.8 %), Hebdomadis (3.37 %), Australis (1.49 %) and Ballum (1.19 %). By using PCR, 33 (15.7 %) out of 210 samples were pathogenic *Leptospira* while no saprophytic *Leptospira* spp. was detected. Partial 16S rRNA gene sequences of *Leptospira* species which were obtained from this study were submitted to GenBank and acquired accession numbers KP313246 and KP313247. Phylogenetic analysis of the nucleotide sequences revealed that species obtained from Katavi-Rukwa ecosystem clustered in the same group with several published pathogenic *Leptospira* specifically *Leptospira interrogans* and *Leptospira kirschneri.* To the best of the authors’ knowledge^,^ this is the first study from Tanzania to confirm pathogenic *Leptospira* in human subjects using genomic typing technique.

**Conclusion:**

These findings provide ultimate evidence of pathogenic *Leptospira* species circulating among agro-pastoralists living in Katavi-Rukwa Ecosystem suggesting that active disease surveillance should be undertaken in order to achieve greater protection of the agro-pastoral communities in Tanzania.

**Electronic supplementary material:**

The online version of this article (doi:10.1186/s12879-016-1588-x) contains supplementary material, which is available to authorized users.

## Background

Leptospirosis is a neglected zoonotic disease of worldwide public health importance which affects humans, domestic animals and wildlife [1]. The disease is caused by different *Leptospira* serovars, which belong to the order of Spirochaetales, family Leptospiraceae, genus *Leptospira* [2]. Leptospirosis causes direct economic burdens to humans such as loss of productivity due to illness, increased treatment costs due to ill health and contributes to poverty among the population in the affected countries [3]. In animals, it causes miscarriages among cattle, stillbirth, loss of milk, infertility, death and related veterinary consequences. According to Global Leptospirosis Environmental Action Network (GLEAN) reports, developing countries including Tanzania have a significant disease burden with more than 500, 000 human cases per year and a mortality rate ranging from 5 % to 10 % [4]. The disease accounts for up to 20 % of febrile illness of unknown origin [5, 6]. Therefore, early detection of the disease in the hosts, prompt treatment, and public awareness raising are among the steps that can be taken to reduce the impact caused by leptospirosis [7].

Despite its impact to humans and animals, leptospirosis is often not considered in the standards of care in Tanzania. The disease is often mistaken for other febrile illnesses such as dengue, malaria, rickettsiosis and enteric fever [8]. This resemblance frequently makes the disease to be misdiagnosed contributing to its status as the most neglected and underestimated disease in Tanzania [9]. Diagnosing leptospirosis in humans is challenging; for example diagnosing using culture technique is not only time consuming but it is also not recommended for clinical diagnosis of leptospirosis in humans [10]. Similarly, the use of antibiotics limits the isolation of the organism in clinical samples. For these reasons, serological testing, specifically the microscopic agglutination test (MAT) remains the test of choice for identification and characterization of the prevalence of *Leptospira* serogroups [10–13]. However, MAT is mainly performed in limited laboratories this is because the test requires considerable expertise [14].

To complement culture and serology; molecular techniques including sequencing techniques have been used by many researchers for the diagnosis and characterization of *Leptospira* species in human and animal samples [2, 15]. The techniques help in identifying epidemiologically important strains and species circulating in a particular geographic area. The identified strains and species may be included in the panel of antigen for serological testing [2]. The sequencing of the *rrs* gene shows that this gene is widely conserved in the genus of *Leptospira,* which originally was grouped into two major clades of pathogenic and non-pathogenic species but now it has been subdivided into three major clades including the intermediate species [16–19]. Although, currently many additional variable genes are targeted for spirochetes classification, the *rrs* gene is still the most widely used for the identification of *Leptospira* species [16].

In Tanzania, there are limited studies on molecular characterization of *Leptospira* species. It is therefore important to know the circulating *Leptospira* spp. in humans in different geographical locations of Tanzania in order to explore the prevalence of *Leptospira* species and provide information to researchers, clinicians, farmers, and policy makers for effective disease surveillance and control. Our research team has previously published data about *Leptospira* serogroups circulating in the Katavi-Rukwa ecosystem using serological assay [1]. This study was undertaken to understand the genetic characteristics of prevalent *Leptospira* species circulating among-pastoralists living in the research naive area of Katavi-Rukwa ecosystem, Tanzania using 16SrRNA based sequence and phylogenetic analysis.

## Methods

### Study area

**Fig. 1:**
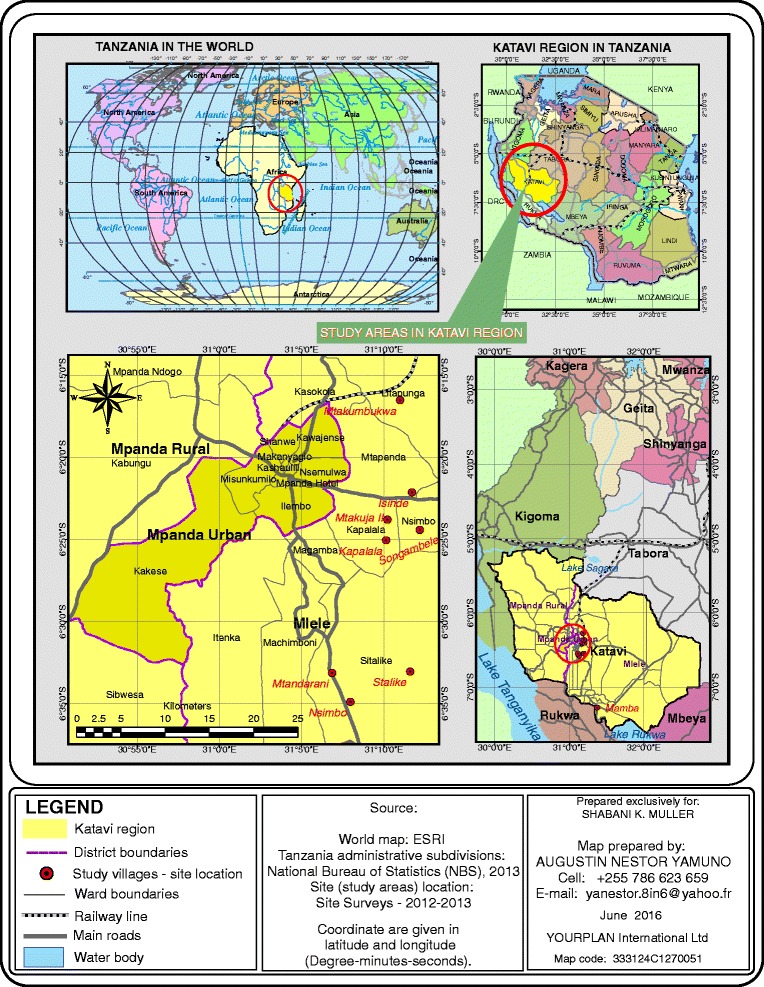
Map of Katavi region showing selected villages where study was conducted including Mpanda and Mlele district

Geographically, Katavi Ecosystem is the research naive area located between latitude 6^0^ 30′ 00” South and longitude 31^0^ 30′ 00” East [20]. It experiences tropical climatic conditions and receives heavy rain fall, which may favor the survival of *Leptospira* spp. In this study, samples were collected from Isinde, Kapalala, Mtakuja II, Mtakumbukwa, Mtandarani, Mamba, Nsimbo, Sitalike and Songambele villages (Fig. 1). All villages belong to Inyonga, Mlele and Nsimbo districts (Fig. 1). Mlele and Nsimbo districts share borders with two protected areas of Katavi National Park and Rukwa Game Reserve.

The main economic activities in this region are agriculture, livestock keeping, honey production, and fishing [21]. Furthermore, there are also a large number of immigrant livestock keepers from Shinyanga and Tabora who are pastoralists and are intensively involved in livestock and farming activities next to the national park and game reserve, hence creating high interaction between humans, domestic animals and wildlife. This interaction exposes the community to a higher risk of zoonotic infections, including leptospirosis.

### Research design

The research design for the present study was a cross-sectional epidemiological study, in which a multistage cluster sampling was carried out as described by Assenga et al [1]. In short, villages were randomly selected as the primary unit, from which a total of 138 households were chosen from the list of agro-pastoralists using random numbers. Members of the selected households were subjected to a random selection to obtain a total of 267 humans who were readily available, regardless of their health status. Samples were collected at different period between August 2013 and September 2014.

### Sample collection

A total of 5–10 ml of blood was collected into a plain and ethylene diamine tetra-acetic acid (EDTA) vacutainer tube (Becton Dickson, Dublin, Ireland) from peripheral veins of each participant for serological and molecular diagnosis respectively. Each sample was labeled using a unique identification number (UID). Samples were transported within 12 h after collection to Mpanda District Hospital (MDH) for processing and storage. Clotted blood in the plain tubes was centrifuged at 3000 *g* for 10 min to obtain clear sera. Sera and blood in EDTA were finally stored in liquid nitrogen and transported to the Sokoine University of Agriculture (SUA) laboratory and stored at -80 °C upon arrival for further use.

### Microscopic agglutination test (MAT)

Ellinghausen McCullough Johson and Harris (EMJH) liquid medium was prepared as per the standard protocols using the EMJH base and Enrichment media (BD Difco TM USA) with 200 μg of 5-fluorouracil per ml of medium. MAT was performed as previously described by Assenga et al. [1]. In short, all sera were tested for antibodies against six *Leptospira* antigens including local serogroups Icterohaemorrhagiae, Ballum, Grippotyphosa, Sejroe and reference serogroups Hebdomadis and Australis

Reference serogroups were kindly provided by Royal Tropical Institute (KIT) *Leptospira* Reference Center, the Netherlands [22]. All sera were serially diluted to 1:10240 in PBS and tested with antigens suspension to establish the antibody titer. MAT titers ≥ 1:160 were scored as positive; titers between 1:20 and 1:80 were scored as exposed to leptospiral infection and absence of agglutination titers was scored as negative [23–26].

### Identification of pathogenic and non pathogenic *Leptospira* by PCR

DNA was extracted from blood using the Purelink Genomic DNA extraction kits (Invintogen, Leuden, and the Netherlands) following manufacturer instructions. The PCR was carried out using reported sets of primers Lept1/Lept 2 and Sapro1/Sapro2 as per the method described by Mgode et al. [22]. These primers can discriminate pathogenic and saprophytic *Leptospira* in animals and environmental samples.

The amplification was performed in a PTC-100 thermal cycler (Mj Research, Watertown, MA, USA) using a Fast *Taq* DNA polymerase (Invitrogen, Leuden, the Netherlands). DNA of pathogenic *Leptospira* including serovars Kenya, (serogroup Ballum) and saprophytic serovars Patoc, (serogroup Semaranga) were used as positive controls for pathogenic and saprophytic *Leptopira* respectively. Nuclease free water was used as negative control.

The PCR amplification conditions for pathogenic and saprophytic *Leptospira* were used. PCR products were visualized after performing agarose gel electrophoresis. Briefly, 0.2 % agarose gel was prepared and pre-stained using Gel Red. Electrophoresis of DNA was performed at 100 V for 30 min followed by visualization of PCR product using a gel documentation system (Applied Biosystem, California, USA).

### Partial DNA sequencing

Ten representative samples of unpurified PCR products were sent for DNA sequencing at Macrogen laboratory (Amsterdam, Netherlands). A 330 base pair of the 16S ribosomal RNA gene was used for confirmation of *Leptospira* spp. The amplified PCR products were purified prior to the sequencing process using a DNA purification kit (Invintogen, Leuden, the Netherlands) as per the manufacturer’s instructions. Direct sequencing was performed in both directions for each PCR product using dideoxy chain termination procedure (Chemistry V3.1; Applied Biosystems, Foster City, CA) and the 3730XL DNA analyzer (Applied Biosystems).

### Statistical analysis

Descriptive analyses were performed with Epi info™ version7 (CDC, USA) and Medcalc software version 12.1 (Osteen, Belgium). Proportions were compared using *χ*2 test. Microsoft Excel was used to compile raw data from field and laboratory works.

### Molecular analysis

Raw sequences data or chromatograms were visualized and trimmed using sequence scanner software version one (Applied Biosystem CA, USA). Then, the forward and the reverse complement nucleotide sequences delimited by forward and reverse primers of several 16S rRNA genes PCR products of *Leptospira* were aligned to obtain a consensus sequence. The consensus sequences of the amplified products were compared with the reference sequences stored in GenBank (www.ncbi.nlm.nih.gov/genbank/) using blast analysis (www.ncbi.nlm.nih.gov) browsed on March 8, 2015. The alignment and sequence similarities were done using ClustalW algorithm implemented within Molecular Evolutionary Genetics Analysis version six (MEGA6); [27] and exported as FASTA file documents. The FASTA document was imported into MESQUITE software version 3.04 [28] for preparation of matrix as Nexus file. The Nexus documents were then uploaded in the online database with accession study URL: http://purl.org/phylo/treebase/phylows/study/TB2:S19240.

### Phylogenetic analysis

Phylogenetic analysis was done by using Molecular Evolutionary Genetics Analysis version six (MEGA6) software [27]. The selected and identical *Leptospira* species representing pathogenic, nonpathogenic, and intermediate species were retrieved from GenBank for comparative analysis. Phylogenetic trees were constructed based on partial nucleotide sequences of 16S rRNA gene of *Leptospira* spp available in GenBank. The tree topologies were evaluated using bootstrap test of phylogeny in the maximum likelihood method and the bootstrap *P*-values were obtained after 1000 replicates of the dataset. The bootstrap consensus tree inferred from 1000 replicates was taken to represent the evolutionary history of the isolates analyzed. Branches corresponding to partitions reproduced in less than 50 % bootstrap replicates were collapsed. The percentages of replicate trees in which the associated isolates clustered together in the bootstrap test were shown next to the branches.

The phylogenetic trees generated from this study were exported as Newick file documents and converted into nexus format using Figtree software version 1.4.2. The tree was deposited in the online Treebase database along with the matrix (http://purl.org/phylo/treebase/phylows/study/TB2:S19240)

## Results

The results obtained from the present study showed that out of 267 samples tested for MAT, 80 (29.9 %) were seropositive (data shown in Additional file 1). The major circulating leptospiral serogroups were Sejroe (15.7 %,), Icterohaemorrhagiae (8.9 %), Grippotyphosa (4.8 %), Hebdomadis (3.37 %), Australis. (49 %) and Ballum (1.19 %).

Positive agglutination titers ranging from 1:160 to 1:1024 were detected for Icterohaemorrhagiae and Sejroe. No titer above 1:160 was detected for serovars hebdomadis. The titers and the number of subjects for each serogroup are shown in Table 1.

**Table 1:** Tested serogroups and its positive agglutination titers

Serogroups	Titers							Total
	1:160	1:320	1:640	1:1280	1:2560	1:5120	1:10240	
Sokoine	2	2	1	9	4	4	2	24
Serjoe	18	9	5	2	5	2	1	42
Hebdomadis	9	0	0	0	0	0	0	9
Grippotyphosa	2	3	3	4	0	0	1	13
Ballum	3	0	0	0	0	0	0	3
Australis	3	0	1	0	0	0	0	4
Total	37	14	10	15	10	6	4	96

### Differentiation between pathogen and saprophytic *Leptospira* by PCR

In the present study, DNA amplification was done separately using two sets of primers Lepat 1/Lepat2 and Sapro1 and Sapro2 in 210 samples that were positive or exposed to *Leptospira* based on MAT results. Thirty three out of 210 (15.7 %) were infected with pathogenic *Leptospira* species; none of the samples was PCR positive with saprophytic *Leptospira*.

### Partial DNA sequencing and phylogenic analysis

**Fig. 2:**
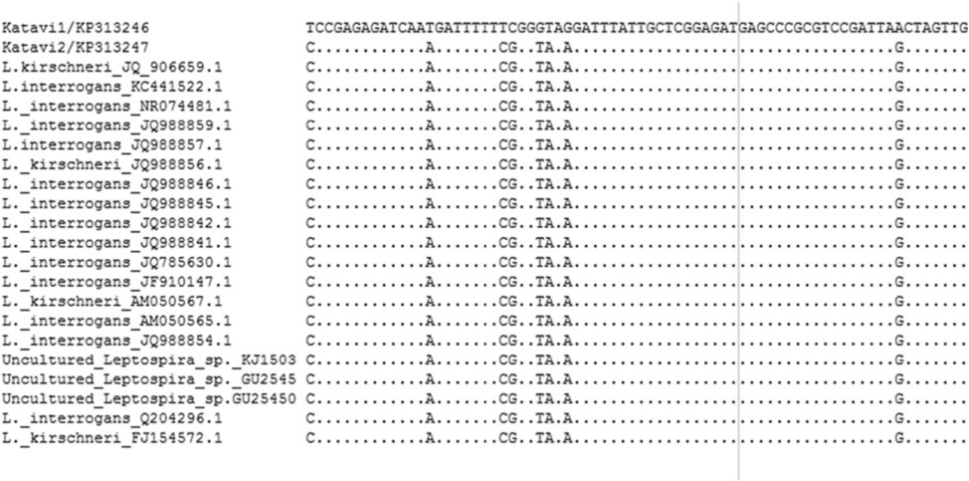
Nucleotide sequence alignment of *Leptospira* species showing nucleotides’ variable sites among pathogenic *Leptospira* species obtained from Katavi and those retrieved from GenBank. KP 313247 is 100 % identical to other *Leptospira* species while KP313246 is 97 %. Dots: represent identical nucleotides in all species

Two PCR products out 10 submitted for sequencing were confirmed as DNA sequences of *Leptospira*. The 16S rRNA partial nucleotide sequences were submitted to GenBank and provided with accession numbers [KP 313246 and KP 313247, GenBank]. Sequences analysis showed 97 % to 100 % identity with published sequences of pathogenic *Leptospira* spp. available in GenBank. Majority of *Leptospira* spp identical to those from Katavi-Rukwa ecosystem were *Leptospira interrogans* and *Leptospira kirschneri.* Similarity to uncultured clone (KJ150300) was also seen (Fig. 2).

**Fig. 3:**
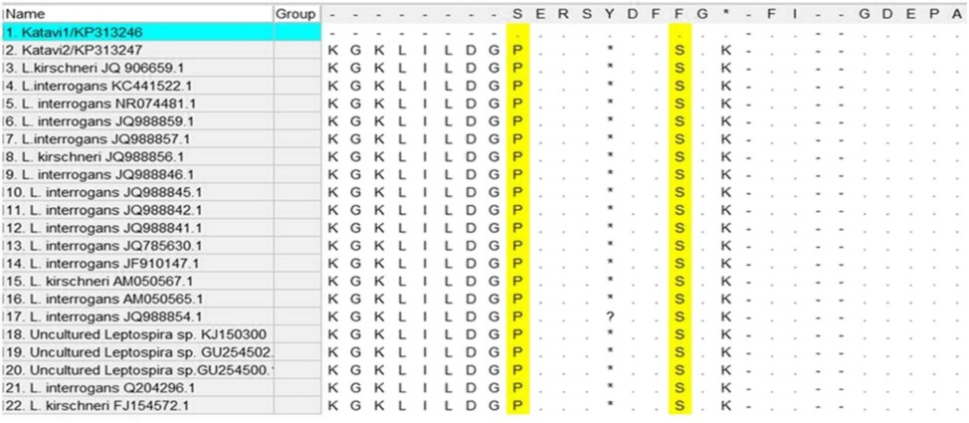
Deduced amino acids showing variable sites of proteins sequences of *Leptospira* from the Katavi-Rukwa ecosystem (highlighted in yellow) and those retrieved from the GenBank (Highlighted in green). Dots represent identical proteins

Comparison between *Leptospira* nucleotide sequences from Katavi (see Additional file 2), showed that KP313247 was 100 % similar to pathogenic *Leptospira* spp retrieved from GenBank while KP313246 was 97 % similar with the same species.. Sequence nucleotides of KP313246 had eight nucleotide variable sites compared to the rest of the sequences included in the alignment. When deduced to amino acid sequences only two variables sites were observed: Serine (S) at position 9 and Phenylalanine (F) at position 16 of KP313246 were replaced by F and S respectively in KP313247 (Fig. 3).

**Fig. 4:**
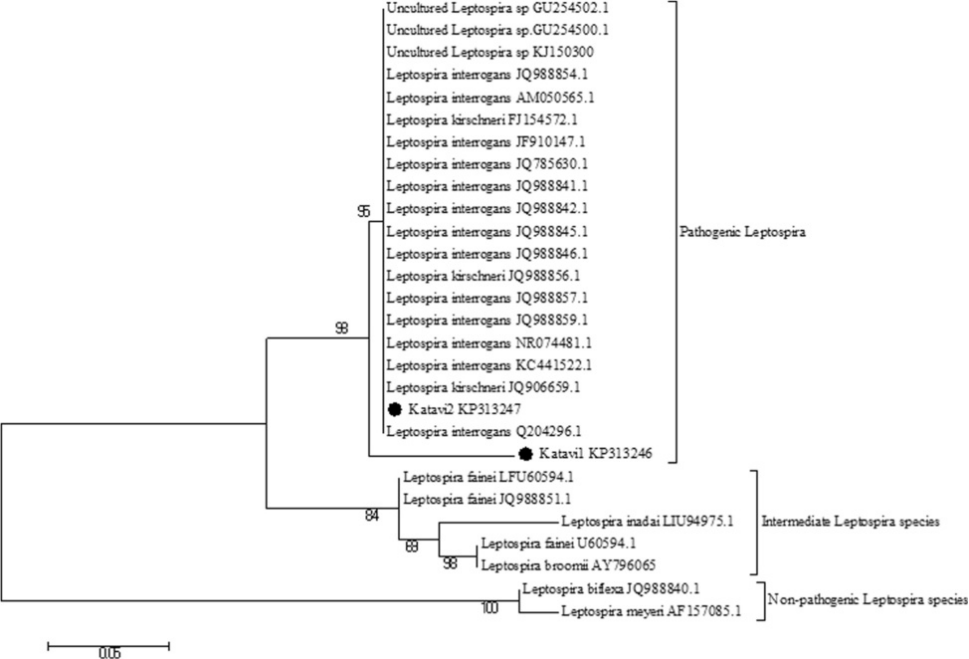
A maximum likelihood phylogenetic tree depicting the relationship between *Leptospira* species obtained from this study (indicated with circles) and those retrieved from GenBank, representing mostly pathogenic species and few intermediate and non pathogenic species. Phylogeny was inferred following 1000 bootstrap replications and values < 50 % were not shown

In this study, phylogenic analysis showed *Leptospira* spp from Katavi-RukwaEcosystem are essentially identical to pathogenic *Leptospira* group. But the Katavi1 KP313246 appeared to be quite variant from the rest of pathogenic *Leptospira* group. (Fig. 4)

## Discussion

The present study was undertaken to understand molecular characteristics of *Leptospira* spp. circulating among agro-pastoral communities of Katavi-Rukwa ecosystem. The results obtained showed that there was an overall positivity of 29.9 % by MAT and 15.7 % by PCR. A previous study conducted in a different research area could not identify any positivity using serology or PCR method [24]. This is probably the first study to confirm pathogenic *Leptospira* spp. in human subjects using genomic typing technique in Tanzania.

The emergence and endemicity of *Leptospira* in the Katavi-Rukwa ecosystem may have resulted from the influx of animal movement to the area and to an increase of pastoral population over the recent years. Leptospirosis diagnosis and management are not routinely conducted in Tanzania including Katavi; this might have led to the endemicity of leptospirosis among the study population. Additionally, leptospirosis may be misdiagnosed and mismanaged as being mistaken for malaria or other pyrexia of unknown origin (PUO) which present with similar clinical presentation that might contribute to a high rate of infection in the study area [8, 29, 30]. MAT results showed multiple sero groups are likely to circulate in the ecosystem whereby serogroups Sejroe and Icterohaemorrhagiae might be considered as highly infectious serogroups in Katavi. These findings are consistent with the findings of earlier studies conducted in other parts of Tanzania, which confirmed serogroups Sejroe, Hebdomadis, Grippotyphosa and Icterohaemorrhagiae as major circulating serogroups [9, 24, 31, 32]. High numbers of rodents, cattle and wildlife are likely to have contributed to high reactivity of these serogroups in Katavi-Rukwa ecosystem as reported previously by our team [1].

High agglutination titers observed in this study may reflect current infection of leptospirosis among agro-pastoralists regardless of their health status. According to WH/OIE, in the absence of paired sera samples, the reactor of high dilution will clearly indicate active and recent infection of leptospirosis conclusively [10, 33, 34]. This observation is essential for future studies.

In contrast with other findings, PCR technique could not detect high number of positivity as did with MAT. Failure to detect high numbers of *Leptospira* DNA in suspected cases could be justified by either the absence of the organism in blood samples or low levels of *Leptospira* DNA yield [17, 35, 36]. PCR detects the presence of bacteria in blood at the onset of disease while MAT detects the rise of *Leptospira* antibody in sera from seven to fifteen days after exposure to the disease. However, it is likely that *Leptospira* spp. from western Tanzania have different molecular characteristics from those reported elsewhere. Further studies should be conducted using urine as a specimen since urine harbors huge numbers of *Leptospira* in apparently healthy individual compared to other specimen [17].

In this study, sequences of two PCR products were obtained from Macrogen Inc Company (Netherlands) where the sequencing technique was done. Type and purification technique used, low yield of *Leptospira* DNA in blood, laboratory errors or contaminations might be the main reasons for obtaining two sequences of pathogenic *Leptospira* out of ten PRC products submitted. However; the obtained sequences were comparable to other sequences available on the GenBank and provided similarities ranging from 97 % to 100 %. This information is useful for public health interventions such as vaccine and development of diagnostic tool.

Based on sequence comparison with published *Leptospira*; the most prevalent species in this study were *Leptospira interrogans and kirschneri* as labeled by their authors. These species are believed to be pathogenic [15]. In our understanding, this is probably the first study to sequence *Leptospira* species circulating in human subjects in Tanzania. Similarity to reference sequence of the uncultured pathogenic clone species (Accession number: KJ150300) observed might provide clues for further source of contamination.

The uncultured clone was isolated from cerebrospinal fluid of a human subject from an environmental sample in Laos; Thailand (KJ150300). This result suggests that transmission of leptospirosis in human subjects in Katavi-Rukwa might be through direct or indirect contact with infected animals, contaminated water, or soil. Also, such leptospiral clones have been responsible for causing outbreaks in Asia. For instance in the year 2000, a dominant pathogenic *Leptospira* clone, which was isolated from a human being, was responsible for the leptospirosis outbreak in Thailand [14].

Additionally, comparison between the two sequences KP313246 and KP313247 showed that KP313246 has more variable nucleotide sites compared to KP313247. However, most of these sites are nonsense codons since only two sites were translated to amino acids sequences. This differences might be contributed by either true mutation or sequencing error. Also, the phylogenetic analyses confirmed that these two strains are identical and belonged to pathogenic group as portrayed in Fig. 4. More studies should be carried out to verify similarities among pathogenic strains circulating in the study area.

In this study, the sequences obtained may not generalize all species circulating in the Katavi-Rukwa ecosystem. However, this study expands our knowledge on the genetic information of *Leptospira* species circulating in Tanzania. Further studies should be carried out in Katavi and other parts of Tanzania for proper documentation of all circulating *Leptospira* species*.*

## Conclusions

In conclusion, molecular techniques have confirmed the presence of pathogenic *Leptospira* species circulating among agro-pastoralists. The presence of *Leptospira* species poses a public health threat to the communities living in the Katavi-Rukwa ecosystem. Active disease surveillance should be undertaken in order to effectively protect agro-pastoral communities against the disease in Tanzania.

## Abbreviations

BLAST, basic local alignment search tool; bp, base pair; DNA, deoxyribonucleic acid; EDTA, ethylene diamine tetra-acetic acid; EMJH, Ellinghausen, McCullough, Johnson and Harris; GLEAN, global leptospirosis environmental action network; KIT, royal tropical institute; MEGA6, molecular evolutionary genetics analysis version six; MAT, microscopic agglutination test; NBS, national bureau of statistics; PCR, polymerase chain reaction; RNA, ribonucleic acid; WHO, world health organization

## Additional files

Additional file 1: Table S1. Prevalence of leptospira antibodies interms of villages in the Katavi region, Tanzania. (DOCX 14 kb) Additional file 2: DNA sequences of pathgenic species identified from this study. (DOCX 14 kb)
