# Supplementary material for: Human leptospirosis in Tanzania: sequencing and phylogenetic analysis confirm that pathogenic Leptospira species circulate among agro-pastoralists living in Katavi-Rukwa ecosystem
Source: BMC Infect Dis. 2016 Jun 10;16:273. doi: 10.1186/s12879-016-1588-x (PMC4902944; doi:10.1186/s12879-016-1588-x)
Supplement: Additional file 1: Table S1. — Prevalence of leptospira antibodies interms of villages in the Katavi region, Tanzania. (DOCX 14 kb) [file 12879_2016_1588_MOESM1_ESM.docx]

**Additional file 1**

|  | **Number of subjects** | **Positive (%)** | **MAT**  **Negative**  **(%)** | **Exposed**  **(%)** |
| --- | --- | --- | --- | --- |
| Isinde | 55 | 19(34.5) | 11(20) | 25(45.4) |
| Kapalala | 10 | 4(40) | 3(30) | 3(30) |
| Mamba | 26 | 3(11.5) | 10(38.4) | 13(50) |
| Mtakuja II | 15 | 8(53.3) | 2(13.3) | 5(33.3) |
| Mtakumbukwa | 46 | 18(39.1) | 3(6.52) | 25(54.3) |
| Mtandarani | 38 | 11(28.9) | 5(13.15) | 22(57.8) |
| Nsimbo | 9 | 2(22.2) | 4(44.4) | 3(33.3) |
| Songambele | 47 | 11(23.4) | 12(25.5) | 24(51) |
| Stalike | 21 | 4(19) | 7(33.3) | 10(47.6) |

**Table S1: Prevalence of *Leptospira* with villages**
