# Supplementary material for: Human leptospirosis in Tanzania: sequencing and phylogenetic analysis confirm that pathogenic Leptospira species circulate among agro-pastoralists living in Katavi-Rukwa ecosystem
Source: BMC Infect Dis. 2016 Jun 10;16:273. doi: 10.1186/s12879-016-1588-x (PMC4902944; doi:10.1186/s12879-016-1588-x)
Supplement: Additional file 2: — DNA sequences of pathgenic species identified from this study. (DOCX 14 kb) [file 12879_2016_1588_MOESM2_ESM.docx]

**Additional file 2**

16S rDNA sequence of two pathogenic strains from this study

1. KP313246 ( GenBank)

TCCGAGAGAT CATATGATTT TTTCGGGTAG GATTTATTGC TCGGAGATGA GCCCGCGTCC GATTAACTAG TTGGTGAGGT AAAGGCTCAC CAAGGCGACG ATCGGTAGCC GGCCTGAGAG

GGTGTTCGGC CACAATGGAA CTGAGACACG GTCCATACTC CTACGGGAGG CAGCAGTTAA GAATCTTGCT CAATGGGGGG AACCCTGAAG CAGCGACGCC GCGTGAACGA TGAAGGTCTTC GGATTGTAAA GTTCAGTAAG CAGGGAAAAA TAAACAACAG CATGTGAACA CC

1. KP313247(GenBank)

TACTTTCCGA AAGGGAAGCT AATACTGGAT GGTCCCGAGA GATCATAAGA TTTTTCGGGT AAAGATTTAT TGCTCGGAGA TGAGCCCGCG TCCGATTAGC TAGTTGGTGA GGTAAAGGCT CACCAAGGCG ACGATCGGTA GCCGGCCTGA GAGGGTGTTC GGCCACAATG GAACTGAGAC ACGGTCCATA CTCCTACGGG AGGCAGCAGT TAAGAATCTT GCTCAATGGG GGGAACCCTG AAGCAGCGAC GCCGCGTGAA CGATGAAGGT CTTCGGATTG TAAAGTTCAG TAAGCAGGGA AAAATA
